# Dr. Carson's Opinions Respecting the Influence of Atmospheric Pressure on the Circulation of the Blood

**Published:** 1827-09

**Authors:** N. Arnott


### PHYSIOLOGY.

### Dr. Carson's *Opinions respecting the Influence of Atmospheric Pressure on the Circulation of the Blood, examined by* Dr. N. Arnott.

Had not Dr. Carson mentioned that he had read as yet only a part of the chapter on the Circulation of the Blood, in my "Elements of Physics," lately published, I should have deemed the appearance of his paper in the last Number of this Journal, questioning some of my conclusions, a proof that I had ill performed my task; but I hope, when he examines the whole,—which perhaps he should have done before venturing to write,—that his opinions will change. I would have trusted to this possibility, and allowed the paper to pass unnoticed, had it not seemed to me to furnish so complete a specimen of the manner in which the *vacuum doctrine*, of which he is accounted the author, has been advocated—the doctrine, viz. that the blood in the veins is moved by suction of the chest, or atmospheric pressure, and not by the propulsive force of the heart, or a *vis a tergo*. I think it will be easy to show that Dr. Carson's present defence of it—in the preparation of which, too, he has had the advantage of knowing all the arguments elicited by the many discussions of the subject during the last year,—involves, or rather consists of, the five following errors:

1st. He assumes as a fact that which is not a fact, and makes his doctrine depend upon it. He says that there is every where around the veins a pressure of parts, nearly such as would be produced by liquid filling the skin as a sack. This assumption is intended to meet the statement in the "Elements of Physics," that no pump or suction can lift liquid through pliant tubes free to collapse like the veins, and that the suction of the chest in ordinary respiration is too weak to lift blood even one inch through tubes of any kind. He then argues that the blood in the veins being thus supported by the surrounding pressure nearly up to the level of the heart, so little suction is required to make it enter the heart, that the veins are not in danger of collapsing. He illustrates the condition of the veins by that of a thin onion-stalk full of water, and immersed in water. Now, where is the supposed pressure on the veins of the back of the hand, for instance, covered as they are only by loose skin,—in old people wrinkled, and almost hanging,—or, indeed, on the superficial veins any where? for in no place has the skin a tension at all resembling that of a sack filled with liquid. The external veins of the leg are nearly as strong as arteries, because of the contained load which they have to support; while deep-seated veins, that are really pressed upon by the flesh, and the veins of the upper parts of the body, are comparatively thin and weak.

2d. He does not perceive that, even if the above assumption were true and admitted, instead of being favourable to his doctrine, it would be an unanswerable proof of the contrary,—that is to say, of the constant action of a *vis a tergo*. For it is evident that no blood could enter the extremities of veins pressed upon as he supposes, unless it were urged by a force greater than such pressure,—viz. greater than the influence of a liquid column reaching from near the heart. Let him try to inject into the bottom of his onion-stalk, while the strongest suction which it will bear is made at its top to assist him. If he were to answer this objection by alleging that the same surrounding pressure acts on the arteries and veins, and becomes the force required, or that the weight of the descending column of blood in the arteries is operative, he would admit the transmission of a pressure through the capillaries from artery to vein, and could not then withhold the same admission in regard to the action or pressure of the heart, which he knows to be sufficient, through open capillaries, to lift the venous blood not only to the heart again, but eight feet beyond it. There is no conceivable state of the veins which does not require the admission of a constantly acting *vis a tergo;* unless they were rendered resisting or rigid tubes, and the chest or heart were a pump strong enough to lift from the feet.

3d. He adduces an acknowledged fact—the tardy flux of blood from a punctured vein,—to disprove the *vis a tergo;* which fact, however, by applying the same reasoning to it, equally disproves his own doctrine; but, when justly viewed, disproves neither. He says, the *vis a tergo* should cause a strong jet from a puncture:—does he not see that the jet should as certainly follow from his assumed pressure round the veins, increasing during expiration? Let him try the effect of a puncture in his onion-stalk. The true explanation of the tardy flux is, that the healthy cut flesh tends to close. A ruptured varix bleeds long enough; and the force with which the blood escapes from a free opening in the veins, is shown by its always rising to even beyond the level of the heart in any tube made to communicate with a vein below the heart.

4th. He adduces as a proof that atmospheric pressure aids absorption, the fact that, if the pressure be taken off from any part of the body by a cupping glass, absorption ceases at the part. The same reasoning would prove that atmospheric pressure helps a man to lift his hand from the mouth of a glass, on which he had rested it; because, if the air be prevented from pressing underneath the hand, as in the well-known pneumatic experiment, the hand is firmly held down to the glass. Nay, the same reasoning would prove that a running horse owes his speed to this all-performing atmospheric pressure; for, if the horse's buttock were brought close to a large opening connected with a vacuum, he would try to gallop off in vain. In the "Elements of Physics" it is shown, and I hope clearly to all, that, as regards absorption in animals, there is always, in parts below the level of the heart, a balance or excess of inanimate pressures opposing it, so that new matter can only be taken in by a vital power capable of overcoming such resistance.

5th. He allows that, when the stream of blood towards the heart is obstructed by a pressure on the veins, by bandage or otherwise, it is the force of the heart, or *vis a tergo*, which makes the veins turgid, and causes the blood to jet strongly upwards from a puncture; yet he asserts that the *vis a tergo* does not act until *after* the veins are turgid. His words are, "Before the action or impetus of the heart and arteries can affect the blood in the veins, it is necessary that the veins be distended to that condition in which they possess the property of rigid tubes." Now, what is the force which *gradually* fills them? No suction can reach them, and the only other imaginable force is the *vis a tergo*. When Dr. Carson makes this assertion, it is as if he said that a pump driving water through a leathern tube only acts on the water when there is some accidental obstruction in the tube.

If it appear to Dr. Carson that these remarks are well founded, he will perhaps excuse the passage in the volume on Physics which imputes his errors rather to the prevailing defect in medical education as regards physics, than to any other cause. That a man of talent like Dr. C., and evidently possessing natural aptitude for physical investigations, should have fallen into such errors, and that he should find followers among medical men, are surely proofs that a defect does exist; and when we reflect that it regards a department of science essential to the explanation of so many of the healthy functions of the animal body, as well as of diseases and remedies, we may judge of the importance of correcting it.

*London; August 13th, 1827.*

